# Child sexual abuse: The significance of the history and testifying on non-confirmatory findings

**DOI:** 10.4102/phcfm.v11i1.1954

**Published:** 2019-06-10

**Authors:** Johanna M. Kotzé, Hanneke Brits

**Affiliations:** 1 Department of Forensic Medicine, University of the Free State, Bloemfontein, South Africa; 2 Department of Family Medicine, University of the Free State, Bloemfontein, South Africa

**Keywords:** child sexual abuse, expert medical witness, sexual assault, non-confirmatory signs, normal examination

## Abstract

**Background:**

Despite numerous studies and publications, there is still a common expectation that a medical assessment can confirm or rule out child sexual abuse (CSA). The truth is that CSA can never be ruled out and can seldom be confirmed on clinical grounds.

**Aim:**

The objective of this article was to suggest which aspects to consider when the expert medical witness in a CSA case needs to explain why CSA can seldom be confirmed and can never be ruled out. The importance of a sound medical and medico-legal history was discussed because the history was generally the only positive ‘finding’ of the assessment of children who have possibly been abused.

**Method:**

Authoritative sources were used to support the explanation of reasons for an absence of corroborative clinical findings in CSA, as defined by the World Health Organization. The authors structured the individual sections by providing a background on which to base the testimony. They then summarised the clinical forensic significance of the information which should be offered in the courts and which should reflect on the court records, to be taken into account in the eventual decision, which will be made by the court.

**Results:**

A guideline was provided for answering questions frequently posed to the expert witness in child abuse cases where there were no positive findings.

**Conclusion:**

A structure for the explanation of reasons for a normal clinical examination when evaluating children who may have been sexually abused may reduce the discomfort of medical witnesses and improve the quality of expert medical testimony.

## Introduction

The courts, parents and caregivers, laypersons and inexperienced healthcare providers commonly expect that a clinical examination will confirm or rule out child sexual abuse (CSA). Healthcare providers may be under the impression that it is their medical responsibility to make a conclusive decision on whether CSA took place. The erroneous perception that healthcare providers have a responsibility to prove whether CSA has happened may cause a sense of loss of control. The insecurity may have the result that healthcare providers are reluctant to become involved in the management of children who may have been sexually abused and thus reluctant to become involved in clinical forensic practice when they cannot make such a conclusive decision.

In reality, the primary responsibility of healthcare providers is to minimise the consequences of CSA by prioritising the best interest of the child regarding emotional and physical health as well as a safe environment.^1^ The burden of proof on whether a child had indeed been sexually abused does not rest with the healthcare providers. The responsibility of healthcare providers as expert witnesses in cases where the clinical examination is non-confirmatory is educational. The courts rely on healthcare providers for the interpretation of medical theory to put a reliable medical examination and opinion on record to interpret the legal significance. This opinion should eventually add value to the comprehensive information that should be taken into account when the courts make the ultimate decision.

A clinical examination commonly shows no confirmatory signs, but the possibility of CSA can never be ruled out. The history, both medical and the account of the alleged abuse, is the kingpin of the conclusion on the likelihood that CSA happened.

## The nature of child sexual abuse

The World Health Organization (WHO) defines CSA as:

The involvement of a child or an adolescent in sexual activity that he or she does not fully comprehend and is unable to give informed consent to, or for which the child or adolescent is not developmentally prepared and cannot give consent, or that violates the laws or social taboos of society.^2^

The medico-legal significance of the proposed definition is that CSA is not restricted to penetrative sexual activity or intercourse but includes non-contact sexual abuse, which will not result in injury and contact non-penetrating sexual abuse, which is unlikely to result in injury. Only penetrative sexual abuse has the potential to cause physical injury to the sexual organs but commonly occurs without it.

Adults and children can sexually abuse other children. The perpetrator is usually in a position of power or there is a power imbalance between the victim and the perpetrator. Child sexual abuse can occur between family members, close relatives and in dating or intimate relationships.^2^

Three types of CSA are often distinguished: (1) non-contact sexual abuse, where the victim may experience threats of abuse, exposure to sexual harassment or visual sexual images; (2) contact sexual abuse involving sexual intercourse; and (3) contact sexual abuse without sexual intercourse, like inappropriate touching or kissing. Manipulation (e.g. psychological, emotional and material), rather than physical force, is usually used. The abuse may occur over time, with more exposure or contact each time, or may be once off.^3^

Barth et al.^4^ reported than non-contact sexual abuse occurs in 17% of men and 31% of women, mixed sexual abuse in 8% of males and 15% of women, contact sexual abuse in 6% of men and 13% of women, and forced intercourse in 3% of men and 9% of women.

## The prevalence of non-confirmatory examination findings in child sexual abuse

When assessing possible CSA, clinical findings differ according to the time between the incident and the medical examination, as well as the selection of the population researched. Van As et al.^5^ reported on children of whom 64% presented within 24 hours (h) and 89.8% within 72 h after alleged incidents of sexual abuse to an emergency surgery unit. In this highly selected group, 48% of children showed lacerations and 16% bruising, erythema or discharge. About 35% of children showed no signs of trauma. A study^6^ performed on legally confirmed cases of CSA in children aged 8 months to 17 years, of whom 42% presented within 72 h after the incidents, found that 63% reported penile-vaginal contact and 34% reported anogenital bleeding at the time of the incident; however, 77% of the examinations were normal or non-specific. Berenson et al.^7^ found that in children who were assessed long after the alleged incidents, findings consistent with previous trauma occurred only in four out of 192 cases.

In 36 pregnant adolescents examined for evidence of penile-vaginal penetration, findings that were suggestive of previous genital penetration were present in 8% of the cases and findings confirming previous genital penetration were present in only 6% of the cases.^8^ Heger et al.^9^ reported normal examinations in 96.3% of children referred for sexual abuse examinations. The participants in their study presented because of disclosed abuse, disclosed sexual penetration, concerning behavioural changes, exposure to abuse, genital abnormalities or medical findings.

A longitudinal study of children who presented with anogenital injuries, pain and bleeding found that 14.6% of the injuries healed completely.^10^ In another study, only 2.2% of sexually abused children who were examined non-acutely had diagnostic physical findings compared with 21.4% of children who were examined acutely.^11^

The clinical forensic significance of these studies is that a large percentage of examinations, evaluating the likelihood of CSA, are normal or non-specific. The longer the interval since the sexual activity, the less the probability of finding abnormalities. Even with early presentation and a clear history of anogenital sexual contact, a significant percentage of sexually abused children show no signs of trauma, even if there is previous documentation of visible injuries and a history of pain and bleeding.

## Importance of the history in child sexual abuse cases

The history is the most important element of the clinical forensic assessment of CSA.^6,7,12,13,14,15^ Non-medical professionals may be under the impression that a medical examination follows a set protocol, irrespective of the direction suggested by the information provided by the history. As a result, lawyers, in particular, may have reservations about the role of the medical history and the history of the alleged incident(s) in the formulation of a medico-legal conclusion. It may be reasoned that healthcare providers search for signs to confirm or support the allegation and may be biased in the process.

The history is, however, important for the medical management in clinical forensic practice as the patient’s health cannot be separated from the legal responsibilities. A good history directs the clinical examination, the fulfilling of health needs, the effective collection of medical and medico-legal specimens, indications to take measures to safeguard a child from further abuse and the assessment of other medical conditions that may explain the clinical picture. Knowledge about the relationship between the alleged abuser and the victim provides an indication of the motivation for and the probable nature of the sexual contact. It is clear that a medical diagnosis or medico-legal conclusion is worthless without a good history.

From a medical point of view, healthcare providers who do not take a good history expose patients to unacceptable health risks. Also, healthcare providers run a medico-legal risk when patients are not managed according to accepted medical guidelines, against which the ‘reasonable doctor’ or healthcare provider is measured.

From a medico-legal perspective, ‘blind’ forensic examinations, done without taking a good history and when the clinical forensic conclusion has to be based only on clinical findings, prevent healthcare providers from using their full arsenal of clinical skills to convey the truth, as a large part of the whole truth lies in the history.

Adams et al.^6^ stated that ‘[*w*]hen the child makes a statement that is clear, consistent, and detailed, the physical examination should not be relied upon to provide the “proof” before proceeding with criminal charges’. Therefore, healthcare professionals should ensure that they gather and document the history just as carefully as they document the clinical examination. A good history with or without clinical evidence of abuse is enough to come to an opinion regarding abuse.

The medical history is not a forensic interview and should not be regarded as an official statement. However, because of its forensic nature, which is additional, but secondary, to the emotional, safety and health needs of a child, the assessment should be conducted according to certain principles of a forensic interview, to prevent laying suggestions and contaminating the value of medico-legal information.^3^

Many cases of CSA are not reported or are reported late. Disclosure is generally delayed, in many cases until adulthood or forever.^16,17^ The relationship between the child and the alleged abuser is relevant because the closer the relationship, the greater the number of instances of abuse, and the longer the duration, resulting in less coercion involved in the sexual abuse and a longer time taken to report the sexual abuse.^17,18^

As stated by Adams et al.,^6^ the assessment of the value of the history from the child relies on three aspects: clarity, detail and consistency of the account. Young children cannot lie elaborately about something they do not know – children seldom exaggerate. Inaccuracies in the narrative account of children will rather be in the form of omission than elaboration. Children cannot fantasise about sexual acts or the use of sexual objects when they have no experience of these things. Clarity of the history means the account of the patient is clear in the sense that healthcare providers do not make assumptions, but let the child explain what is meant.^16,18^ Children have a limited vocabulary; they may over-extend or under-extend the meaning of words. They may use strange terms for the anogenital region and commonly do not have terms for the genitalia.

Eliciting a non-leading explanation of the meaning a child attaches to certain words may add to the degree of clarity.^19^ One aspect which recently received attention is the ability of children to distinguish between genital and vaginal penetration and their perception of the word ‘inside’, against the background of their limited understanding of their anatomy and their lack of sexual experience.^11^

Young girls do not have sufficient knowledge of anatomy or experience of sexual activity to be able to differentiate between vaginal penetration, labial penetration and inappropriate touch and the ability to describe the actions. The healthcare provider should further assess the detail contained in the history. The more information a child provides, the more likely it is that the account is credible. Sensory information such as smell, taste, touch, sound (including what was said at the time), what was seen and description of place, time and circumstances adds detail which the child could unlikely have learnt from watching explicit material on the television or videos, or from being instructed by a third person(s) to make false allegations.^6^

The third aspect to assess is the consistency of the accounts given by the child at different times. Consistency may be suggested by information from the police, from medical records and from referrals by social workers and healthcare providers, but it is generally not evaluated by healthcare providers, as information thus required is hearsay.

The medico-legal significance of a history of CSA is that healthcare providers, when evaluating a child, who has possibly been sexually abused, rely heavily on the history.^6,7,12,15^ A clear, detailed and consistent history suggests that CSA has probably occurred.^6^ Questions regarding the possibility of laying suggestions or making assumptions when taking the history may arise in court. Be prepared to answer them.

## Reasons for the lack of confirmation of child sexual abuse on clinical examination

It is clear that a large percentage of sexually abused children have no confirmatory clinical signs, even when penetrative sexual abuse has taken place. The expert clinical forensic witness should be able to explain the reasons why a clinical examination can seldom confirm and never negate the occurrence of CSA. The reasons for the lack of abnormal physical findings are multifactorial.

### Tissue factors

Human tissue has an intrinsic resilience. Only when the force applied is greater than the ability of the tissue to maintain its integrity, visible injuries result. Thus, the absence of visible injuries does not exclude forces that work in on the tissue. Furthermore, human tissue can repair with or without scar tissue formation. The elasticity and the resistance of tissue against injury differ between individuals and different types of tissue. Age, certain medical conditions, certain medications and blood supply to tissues are some of the reasons for individual differences. Anogenital injuries in children heal fast because of a good blood supply.^16,20,21^ Most anogenital injuries heal without residual damage, and this holds true for perianal injuries, hymenal and non-hymenal injuries.^10,20,22^

The influence of oestrogen on genital tissues further increases its resilience. There is an influence of the maternal oestrogen on the genital tissues of infants in the first 18 months to 3 years.^23,24,25^ During this time, the hymen shows characteristics similar to that of the adolescent hymen. During puberty, endogenous oestrogen levels increase to prepare the female body for reproduction. The effect of oestrogen on genital tissues increases its resilience by increasing elasticity, particularly the elasticity of the hymen.^8,22,26^ The increased elasticity commonly prevents the formation of tears. The absence of tear formation prevents the formation of clefts, a structural change that may be visible after healing of a tear. The folds of a redundant or oestrogenised hymen may obscure clefts. The vaginal epithelium, under the influence of oestrogen, changes from single cell columnar epithelium to a thicker multilayer squamous epithelium, which decreases the probability of visible injury.

The clinical–forensic significance of the physiology of tissue is that contact may occur without injury, the injury may heal, healing may occur fast and commonly does not leave visible signs. The larger the interval between the contact and the examination, the less the chance of residual clinical signs. The influence of oestrogen on the genital tract of girls during puberty and infancy further minimises injury through increased elasticity and the formation of more resilient epithelial tissue.

### Anatomical factors

The female genital anatomy consists of external structures (labia majora, labia minora and the enclosed vestibule) and internal structures (hymen, vagina, uterus and adnexa). The slightest penetration into the female genitalia that is between the labia constitutes genital penetration.

Although penetration may extend beyond the external genitalia through the hymen and into the vagina, this is not a prerequisite for a conclusion that genital penetration has taken place. It is unlikely that penetration of the external genitalia might show physical signs, other than transient redness or abrasion, which heals fast.

The anal canal is obliterated by two sphincters. When the external sphincter is constricted, the perianal area forms folds. These folds or rugae can expand to enlarge the opening for passing stools when the sphincter relaxes. Thus, the anus has a significant ability to dilate, and objects can penetrate from the outside without causing injuries. The mouth has a large cavity and can be penetrated without visible injury.

The medico-legal significance of the anatomy of the sexual organs is that the female genitalia, the anus and the mouth can be penetrated without any visible injuries.

### Lubrication

Perpetrators of penetrating CSA may apply a lubricant to the anogenital area in the form of commercial products, saliva or other substances. Menstrual fluid may provide a degree of lubrication. Physiological lubrication, such as a discharge, because of the influence of oestrogen of infants and adolescents and the lubrication caused by the human sexual response, which is physiological and may be present in the absence of enjoyment of the sexual encounter, may account for the absence of anogenital injuries.^27^

The clinical forensic significance of the possibility of lubrication is that, when the tissue is ‘slippery’ because of physiological or applied lubrication, the probability of injuries decreases.

### Perpetrator factors

More often than not perpetrators are family members.^3^ A study on forensic evidence collection reported that children knew the perpetrators in 83% of cases.^28^ Intra-familial perpetrators have easy access to the victim; the victim trusts the perpetrator and may be in a loving relationship with the person.^17,18^

Perpetrators may create time and space to groom children sexually, to gain access to the children, to prevent disclosure and to prevent caregivers from believing the children if they disclose sexual abuse. This group of sexual predators generally does not cause injury or leave other forms of evidence. They may control the children with gifts, secrecy, bribery or threats and not with force.

Perpetrators of CSA have different motivations for the behaviour, which do not differ much from those of perpetrators of rape of adults. The most common motivation of perpetrators sexual offences is to reassure their power. Most are timid and want to believe that their victims enjoy the experience.^29^ A smaller percentage is categorised as exploitative rapists, anger rapists and sadistic rapists. The last three categories are dangerous and may be impulsive and opportunistic, motivated by displaced anger and rage, or motivated by the enjoyment of the suffering of victims.

Certain perpetrators of CSA prefer sexual gratification from children (preferential child molesters), and others prefer sexual gratification from adults, but for various reasons they use children as substitutes (situational child molesters). Both categories contain perpetrators in a continuum of relatively benign to utterly dangerous.

The medico-legal significance of the classification of perpetrators is that most perpetrators avoid hurting children during sexual abuse.

### The mechanism of the child sexual abuse

Perpetrators may also refrain from using force. When the victim is non-resistant, force is not imperative to obtain the desired result. The definition of the WHO includes the probability that abuse may take place without force.^3^

During penetration, the penis may be positioned postero-anteriorly between the labia majora and the labia minora into the vestibule, and the movement is directed in this plane with the ventral aspect of the glans, frenulum and shaft of the penis in contact with the vestibule and the lateral aspects partly in contact with the labia. The same mechanism may apply to penetration with a finger or an object.^30^

The clinical forensic significance of the nature of the mechanism of penetration of the sexual organs is that penetration need not only extend into the vagina or the anus, but may also be interlabial (vulvar) or intergluteal.

### Lack of DNA evidence or semen

Collection of biological evidence from the bodies of pre-pubertal children, especially in those younger than 10 years, particularly more than 24 h after exposure, is of limited value.^28,31,32,33,34^ When DNA is recovered in CSA cases of children younger than 10 years, it is more likely from the clothing or bedding than from the body.^28,31,34^ In cases when DNA was found on the bodies of children, the children were generally pubertal.

The genital tracts of pre-pubertal girls are immature. The vagina is short. Fornices are absent because the cervix is flush with the uterus. Intravaginal rugae only develop at puberty, and the absence thereof renders the vaginal wall smooth and without recesses. Epithelial shedding is rapid in children, and this further diminishes the possibility of DNA evidence.

The medico-legal significance is that the probability of retrieving donor DNA from the bodies of pre-pubertal children after sexual contact is slim. Material containing DNA is more likely to be present on linen and clothing. The yield is higher in adolescents.

### Examination and examiner factors

Lack of resources, such as adequate lighting, toluidine blue tissue stain to highlight injuries and a colposcope may all lower the sensitivity and specificity of clinical examinations.^35,36,37,38,39^ Lack of technical support has the additional consequence that peer review may be unattainable.

Examinations are often technically difficult because of fear and resultant tension of the child. If the child is not relaxed, visualisation of the anogenital structures may be obscured. Technical challenges may influence findings. If a child refuses an examination in a case where there are no medical indications for clinical examination, the reluctance of the child should be respected to avoid secondary traumatisation.^10^ Although this is only relevant to a small percentage of children, it may render an examination to not be in the best interests of a particular child. Examinations under anaesthesia with no medical indications and without health benefits for children are also not in the best interests of children.

Redundancy of the oestrogenised hymen also increases technical challenges of the medical examination by forming folds and making it difficult to visualise the edge of the hymen and detect clefts, unless when making use of special examination techniques. The examiner may also account for inaccuracy in diagnosis because of inexperience or human error, or a difference of opinion.^15,40,41,42,43^

The opportunity for peer review by experts is limited by the scarcity of experience in the field and also by the dangers of sending sensitive images of children over the Internet.

The forensic significance is that examinations may be compromised in certain cases if the examiners are not sufficiently equipped or experienced, or the examination is technically difficult.

### False allegations

One cannot completely rule out false allegations as the reason for a normal examination. However, false allegations are uncommon. In one study, 8% of 576 children made false allegations.^44^ Adolescents may make more false allegations than children younger than 6 years, where the rate of false allegation is 2%.^45^ If the history of sexual activity by a young child is detailed, it is unlikely that it is not true. As mentioned previously, it is unlikely that children can lie or exaggerate when they have no experience of sexual activity.^18^

The medico-legal significance of the probability of false allegations is that false allegations are possible, but uncommon, particularly in pre-pubertal children. A good history may indicate the credibility of an allegation.

## Key learning points

The following are important pointers to take into consideration in the assessment of any CSA case:

- The clinical assessment of children who may have been sexually abused can seldom confirm and never exclude CSA.
- The account of sexual activity given by the child is of the utmost importance in the assessment.
- Child sexual abuse, per definition, includes actions that do not result in injury.
- The anatomical definition of female sexual penetration includes vulvar penetration, which is unlikely to result in injury.
- Human tissue has intrinsic characteristics, which prevent acute injuries and visibility of healed injuries.
- Physiological and applied lubrication reduce the probability of injuries.
- The majority of perpetrators avoid causing injury by seducing instead of forcing the child victims.
- Perpetrators avoid injuries by controlling the manner of the anogenital and oral penetration. Penetration of the external genitalia will be unlikely to cause injury.
- The probability of obtaining DNA evidence is low in pre-pubertal children.
- The examination may be technically compromised because of lack of resources or reluctance of the patient.
- Examiners can make human errors.
- False allegations are uncommon, but possible.

## Conclusion

A structure for the explanation of reasons for a normal clinical examination when evaluating children who may have been sexually abused may reduce the discomfort of medical witnesses and improve the quality of expert medical testimony.
